# Transient lower extremity lymphedema following COVID-19 vaccination: A case report

**DOI:** 10.1097/MD.0000000000028092

**Published:** 2021-12-03

**Authors:** Jae-Ho Chung, Sung-Min Sohn, Hyun-Joon Yoo, Eul-Sik Yoon, Seung-Ha Park

**Affiliations:** a Department of Plastic and Reconstructive surgery, Korea University Anam Hospital, Seoul, Korea; b Department of Physical Medicine and Rehabilitation, Korea University Anam Hospital, Seoul, Korea.

**Keywords:** COVID-19, lymphedema, vaccination

## Abstract

**Rationale:**

Complications from COVID-19 vaccines have yet to be sufficiently analyzed because they are rapidly approved without long-term data. In particular, there are no case reports of lymphedema in a healthy patient following vaccination. Herein, we report a patient who underwent transient lymphedema after vaccination with BNT16b2.

**Patient concerns:**

A 79-year-old woman with pitting edema in both lower legs after administration of a second dose of Pfizer vaccine was referred to our clinic. In the absence of clinical evidence of swelling during the laboratory evaluation, we suspected deep vein thrombosis. However, ultrasonographic findings revealed no evidence of venous thrombosis or varicose veins.

**Diagnosis:**

On the basis of lymphoscintigraphy, the patient was diagnosed with transient lymphedema with decreased lymphatic transport in both lower extremities.

**Intervention:**

The patient received intensive physiotherapy, including complex decongestive physiotherapy and pneumatic pump compression, to improve the lymphatic circulation. Furthermore, the patient was trained to apply a multilayer compressive bandage to the lower extremities.

**Outcomes:**

At 2 months follow-up after rehabilitative treatment, the patient's symptoms improved without recurring lymphedema.

**Lessons:**

In the absence of clinical evidence of swelling during laboratory evaluation or ultrasonographic investigations suggesting deep vein thrombosis, we should consider the possibility of lymphatic disorders.

## 1 Introduction

Since the coronavirus disease 2019 (COVID-19) outbreak began in December 2019, approximately 220 million patients have been diagnosed to date, with an estimated 4.5 million deaths. Efforts to lower the rate of infection entailed vaccination with COVID-19 vaccines such as BNT162b2 (Pfizer-BioNTech) or ChAdOx1 nCov-19 (AstraZeneca). However, complications from these vaccines have yet to be sufficiently analyzed because they are rapidly approved without long-term data.

Lymphedema is a well-known disease entity that causes lymphatic fluid stasis, tissue fibrosis, and hypertrophic fat, resulting in skin ulceration and infections.^[1]^ Once symptoms occur, it is very difficult to treat and can negatively affect the quality of life of patients. Approximately 99% of these patients develop secondary lymphedema, which most often occurs after lymph node dissection following cancer resection.^[2]^ In the current publication, however, we report a patient who underwent transient lymphedema after COVID-19 vaccination with BNT16b2 and ChAdOx1 nCov-.19. This case report was approved by the institutional review board of our facility (protocol number K2021-2198).

## 2 Case description

A 79-year-old female patient with no history of underlying diseases visited the clinic because of pitting edema in both lower legs. Physical examination revealed no motor or sensory impairment in both lower extremities, but showed limited movement due to leg swelling. The only positive history element was that she had received a second dose of BNT16b2 1 week before the clinic visit.

The patient was initially suspected of having heart failure or renal disease, but there were no abnormal findings on echocardiography. Regarding laboratory evaluation, her serum BUN was 13.6 mg/dL and serum creatinine level was 0.59 mg/dL, suggesting no progression of renal disease. In addition, there were no specific abnormalities in urine analysis or other blood parameters, including thyroid function test results. As she was an elderly patient and visited a clinic with sudden symptoms of lower extremity edema, we suspected the possibility of deep vein thrombosis (DVT). Thus, additional Doppler sonography was performed to assess the patient's vessel status. However, ultrasonographic findings revealed no demonstrable evidence of venous thrombosis along the bilateral common femoral, superficial femoral, popliteal, posterior tibial, peroneal, and anterior tibial veins. In addition, no demonstrable enlargement of venous structures in both lower extremities was detected, suggesting no definitive evidence of varicose veins.

**Figure 1:**
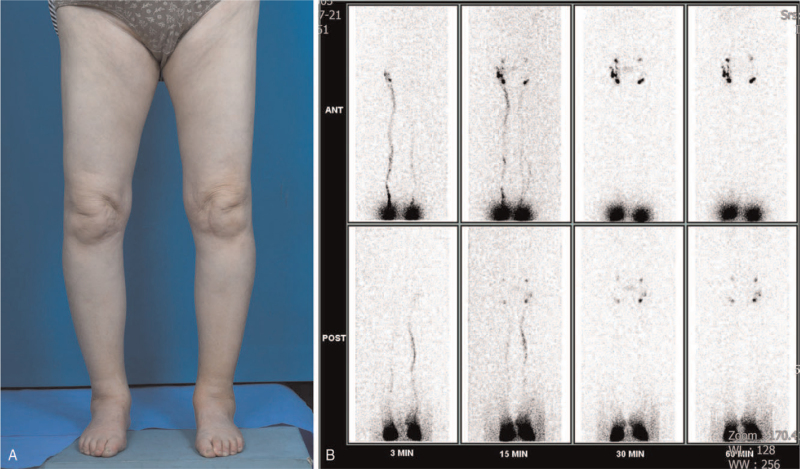
Clinical photograph of patient. (A) A 79-year-old woman manifested bilateral lower extremity swelling 1 week after COVID-19 vaccination. (B) Lymphoscintigraphy showed a decreased lymphatic transport in both lower extremities, especially on the left side.

Next, the patient received a related work-up considering the possibility of lymphedema. Lymphoscintigraphy was performed after injection of technetium (Tc)-99m phytate colloid into the web space of both feet, and serial images of both lower extremities were obtained at 3, 15, 30, and 60 minutes (Fig. 1). In the early phase, the main lymphatic vessel of the left lower extremity was weak compared with the contralateral side. In the late phase, there was no significant dermal back flow, but her left inguinal lymph node activity was weak. The patient was referred to the rehabilitation clinic with suspected decreased lymphatic transport in both lower extremities (worse in the left). She received intensive physiotherapy, including complex decongestive physiotherapy (CDP) and pneumatic pump compression (PPC) to improve the lymphatic circulation. Furthermore, the patient was trained to apply a multilayer compressive bandage to the lower extremities. In addition, we prescribed an oral medication, Entelon 150 (Hanlim Pharm Co., Yongjin, Korea), which consists of grape seed extract, known to improve lymphatic fluid stasis.

The patient's symptoms improved in the first month, showed a pattern of edema reduction when waking up in the morning, and toe discoloration was also improved. Finally, the rehabilitation treatment was terminated after 2 months without recurring symptoms of lymphedema.

## 3 Discussion

Two years since the first detection of this novel severe acute respiratory syndrome coronavirus 2 (SARS-Cov-2) named COVID-19, the public health threat has yet to be resolved. COVID-19 is associated with a wide spectrum of clinical manifestations, the most notable of which is increased prevalence of venous thromboembolism (VTE), including DVT.^[3]^ Recent reports suggest that not only the disease per se, but also vaccines can induce cerebral venous thrombosis, portal venous thrombosis, and DVT, which are serious concerns.^[4,5]^

If a patient has no history of underlying diseases or abnormal findings on laboratory examination, visits the clinic due to intractable leg pain or swelling, DVT can be considered first as a diagnosis, especially if the patient has a history of COVID-19 or vaccination. However, in the absence of specific findings on venous compression ultrasound or abnormal findings in tests such as D-dimer, it may be difficult to treat the patient's symptoms.

To the best of our knowledge, there are no case reports of lymphedema following COVID-19 vaccination. To date, we have identified 3 patients with pitting edema caused by a lack of lymphatic transport in patients with no specific cause or abnormalities detected in other examinations. All patients developed symptoms with pitting edema in the lower legs 1 to 2 weeks after vaccination and were referred to our clinic because of undetected. abnormalities in other tests. After confirming decreased lymphatic activity on lymphoscintigraphy, the patients received rehabilitation via CDP and PPC combined with compressive bandages. All patients showed improvement in transient lymphedema within 2 months (Table 1).

**Table 1:** Patient demographics.

No.	Age	Sex	Vaccine type	BMI	Underlying disease	Smoking	Lymphedema side	Onset after vaccination	Treatment	Duration of recovery
1	79	F	Pfizer-BioNTech	23.9	None	No	Both	1 wk	CDP, PPC, Bandage	2 mo
2	79	M	Pfizer-BioNTech	25.8	None	Ex-smoker	Both	1 wk	CDP, PPC, Bandage	2 mo
3	68	F	AstraZeneca	27.7	Hypertension	No	Left	2 wks	CDP, PPC, Bandage	1 mo

Until now, the Centers for Disease Control and Prevention (CDC) and related organizations such as lymphatic education and research networks have only been interested in vaccinating patients with lymphedema.^[6]^ However, in several patient communities, it is often found that patients with no history of underlying disease complained of mild lymphedematous symptoms involving the lower extremities after vaccination.

All currently available COVID-19 vaccines are administered intramuscularly for their efficacy. Both Moderna (mRNA-1273) and Pfizer-BioNTech (BNT162b2) vaccines use lipid nanoparticles (LNPs) as mRNA carriers. The AstraZeneca (ChAdOx1 nCov-19) vaccine uses adenovirus vectors. The effectiveness of these vaccines depends on their delivery to lymph nodes through dendritic cells. Some antigens may be transferred directly to lymph nodes.^[7]^ In this process, some patients are susceptible to vaccine-induced lymphadenopathy, especially those involving afferent lymphatics or lymph nodes.^[8]^ Therefore, the CDC recommended that COVID-19 vaccine should be administered on the opposite arm or leg in patients at risk of lymphedema.

Our patients showed limited swelling patterns below the knees, and even if only 1 side occurred, there was no difference in circumference from the other side. However, they complained of inconvenience, such as a feeling of heaviness, discoloration of the toe, and limited movement due to swelling of the legs. This seems to be caused by the structural differences between the upper and lower extremities. Because the lower extremities are much larger and more gravity-dependent than the upper extremities, it is thought that symptoms manifest in the distal lesion of the lower extremity, which is most affected by gravity.^[1]^ However, as most of the symptoms are mild, compression and physical therapies can lead to substantial improvement in a short period of time without the need for surgical treatment.

In summary, this case report suggests that patients who have been vaccinated against COVID-19 can present with swollen legs without any specific cause. If their legs swell without any specific cause, the possibility of DVT should first be considered. However, in the absence of abnormal findings on ultrasonography, transient lymphedema due to dysfunction of the lymphatic system can be considered. If lymphoscintigraphic findings are confirmed, because most of the symptoms are mild, improvement is seen via short-term rehabilitation therapy alone. In addition, in patients at risk of lymphedema or a history of lymph node dissection due to previous cancer resection, COVID-19 vaccines should be injected on the opposite arm or leg.

## Author contributions

**Conceptualization:** Chung JH.

**Data curation:** Chung JH, Sohn SM

**Original draft:** Chung JH, Yoo HJ

**Writing – review & editing:** Yoon ES, Park SH
